# Prognosis of Multifocal Papillary Thyroid Carcinoma

**DOI:** 10.1155/2013/809382

**Published:** 2013-12-30

**Authors:** Sheng-Fong Kuo, Shu-Fu Lin, Tzu-Chieh Chao, Chuen Hsueh, Kun-Ju Lin, Jen-Der Lin

**Affiliations:** ^1^Division of Endocrinology and Metabolism, Department of Internal Medicine, Chang Gung Memorial Hospital, Chang Gung University, Taoyuan 333, Taiwan; ^2^Department of General Surgery, Chang Gung Memorial Hospital, Chang Gung University, Taoyuan 333, Taiwan; ^3^Department of Pathology, Chang Gung Memorial Hospital, Chang Gung University, Taoyuan 333, Taiwan; ^4^Department of Nuclear Medicine; Chang Gung Memorial Hospital, Chang Gung University, Taoyuan 333, Taiwan

## Abstract

This study was to investigate the clinical features and therapeutic outcomes of multifocal papillary thyroid microcarcinoma (PTMC). A total of 2,418 papillary thyroid carcinoma (PTC) patients had undergone thyroidectomy in one medical center between 1977 and 2010. There were 483 (20.0%) diagnosed with multifocal PTC. The percentage of multifocal PTC was higher in PTMC patients (22.0%) than in non-PTMC patients (19.5%). Demographic and clinical characteristics of PTMC and multifocal PTC in PTC patients were traced. Multifocal PTC patients presented with smaller tumors at an older age, and a higher percentage underwent total or complete thyroidectomy. These patients also showed a higher incidence of postoperative disease progression than did unifocal PTC patients. Comparison of 483 patients with multifocal PTMC and non-PTMC tumors showed a higher incidence of postoperative disease progression in patients with non-PTMC; otherwise, there was no statistical difference in disease-specific and total mortality between these two groups. In conclusion, the incidence of multifocal PTMC was not lower than that of non-PTMC, and postoperative therapies were necessary for both multifocal PTMC and non-PTMC patients.

## 1. Introduction

Over the past 2 decades, numerous clinical reports have noted an increased incidence of both papillary thyroid carcinoma (PTC) and papillary thyroid microcarcinoma (PTMC) [1, 2]. Although most PTC patients show a good prognosis on initial followup, the mortality rate for PTC after long-term followup is 5–10%. Multifocal PTC has higher recurrence than that of unifocal PTC [3, 4] and mainly results from radiation exposure, genetic mutation, and/or intrathyroid spread [5]. Differences in therapeutic intervention and long-term followup between patients with multifocal PTC and PTMC have generated considerable controversy; however, these alternative approaches are necessary in order to provide appropriate treatment. The purpose of the present study is to investigate the clinical features, therapeutic outcomes, and percentage of multifocal PTC in both PTMC and non-PTMC tumors in areas unexposed to radiation. Additionally, the percentages of PTMC and multifocal PTC found during recent decades were retrospectively analyzed.

## 2. Subjects and Methods

For our retrospective analysis, we collected the records of 2,418 PTC patients who had undergone thyroidectomy at the Chang Gung Medical Center (CGMC) (Linkou, Taiwan) between 1977 and 2010. All of the patients were followedup until the end of 2011 and were staged in accordance with the tumor-node-metastasis (TNM) staging criteria proposed by the Union for International Cancer Control (6th edition) [6]. Patients who were not followedup for at least 1 year, as well as those who underwent an initial thyroid surgery at a different hospital, were excluded from the study.

Preoperative thyroid ultrasonography and fine needle aspiration cytology (FNAC) examinations were performed for 1,908 of 2,418 patients with thyroid nodules [7]. Patients with cytologically proven malignancy or suspected malignancy were advised to undergo thyroidectomy. Total thyroidectomy was performed for 1,999 of 2,418 (82.7%) patients. Out of all 2,418 cases, 483 displayed multifocal PTC. Of these multifocal PTC patients, 454 (94.0%) underwent total thyroidectomy. Following total thyroidectomy, 1,809 patients received postoperative thyroid remnant ablation, radioactive iodine (^131^I) therapy, and long-term followup at CGMC, with the exception of the low-risk T1a without metastasis group. Ablation of thyroid remnant was performed 4 to 6 weeks after surgery, with an ^131^I ablation dose of 1.1–3.7 GBq (30–100 mCi). Cases in which foci of ^131^I uptake, cytological findings, or histological findings indicated extension beyond the thyroid bed were classified as postoperative progressive disease. In these cases, patients were given higher ^131^I therapeutic doses (3.7–7.4 GBq (100–200 mCi)) that were repeated at 6- to 12-month intervals. Permission was obtained from the Institutional Review Board (IRB) and ethics committee of CGMC for a retrospective review of the medical records of study subjects. The IRB waived the requirement for obtaining informed consent. Confidentiality of the research subjects was maintained in accordance with the requirements of the IRB of CGMC.

Pathological classification was performed for all patients according to World Health Organization guidelines [8]. The largest tumor size during the first thyroid surgery was recorded. Patients were categorized as PTMC if the largest tumor diameter was ≤1 cm; otherwise, patients were categorized as non-PTMC. As per our previous study, multifocal PTMC was defined as two or more tumor sites with a diameter ≤1 cm [9]. Noninvasive radiologic and nuclear medical studies were selected based on clinical indications for each patient and included the following: chest radiography, computed tomography, magnetic resonance imaging, bone scan, thallium-201 scan, and fluoro-18-deoxyglucose positron-emission tomography. Postoperative persistent disease status was defined as a persistent local regional tumor or distant metastases as detected by noninvasive methods during the first postoperative year. At the end of 2011, patients were categorized as progression free (PF) on the basis of negative results on ^131^I whole-body scan (WBS), absence of visible tumor outside the neck area, and absence of local or distant metastases in noninvasive examinations. Clinical postoperative progression of PTC was defined as the presence of cytologically or pathologically confirmed lesions or detectable stimulated thyroglobulin (Tg) levels (>1.2 ng/mL).

Admission records were reviewed for the following data: age, gender, primary tumor size, ultrasonographic findings, FNAC results, thyroid function before surgery, surgical methods, histopathological findings, TNM stage, serum Tg levels 4 to 6 weeks after surgery, Tg antibody titers, therapeutic ^131^I scanning results, ^131^I accumulated dose, postoperative chest radiography findings, clinical status for the analysis of distant metastases vianoninvasive radiologic and nuclear medical studies, treatment outcomes, cause of death, and survival status.

Data are expressed as the mean ± standard error of the mean. Univariate statistical analysis was performed to determine the significance of various factors according to the Kaplan-Meier method and the log-rank test [10]. A *P* value <0.05 was considered statistically significant. Survival rates were calculated according to the Kaplan-Meier method and compared with the Breslow and Mantel-Cox tests.

## 3. Results

**Figure 1:**
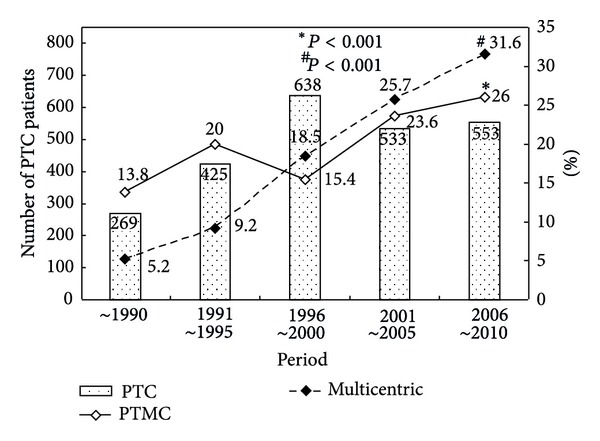
Number of papillary thyroid carcinoma (PTC) patients and percentage of papillary thyroid microcarcinoma and multifocal PTC during different periods.

For all 2,418 PTC patients, the number of total PTC, PTMC, and multifocal PTC cases in different periods is illustrated in Figure 1. The percentage of multifocal PTC and PTMC increased 6.1-fold during the period before 1990 and 1.9-fold during the period between 2006 and 2010. Among the 483 multifocal PTC patients, 108 presented with multifocal PTMC (22.4%) (Table 1). A significantly higher percentage of non-PTMC patients underwent total or complete thyroidectomy (96.8% non-PTMC versus 84.3% PTMC). There were no differences in the gender or mean age of patients between groups. The incidence of TNM stage I was higher for PTMC tumors than for non-PTMC tumors; in addition, the non-PTMC group had a higher rate of postoperative disease progression (Table 1). Otherwise, there were no statistical differences with regard to thyroid cancer disease-specific and total mortality between the 2 groups after a follow-up period of 6.1 years.

To identify risk factors for postoperative progression, clinical and demographic information for multifocal PTC cases was grouped by postoperative progression status and analyzed (Table 2). The results indicated that male gender, larger tumor size, advanced TNM stage, and high postoperative Tg level were significant factors for postoperative progression.

**Figure 2:**
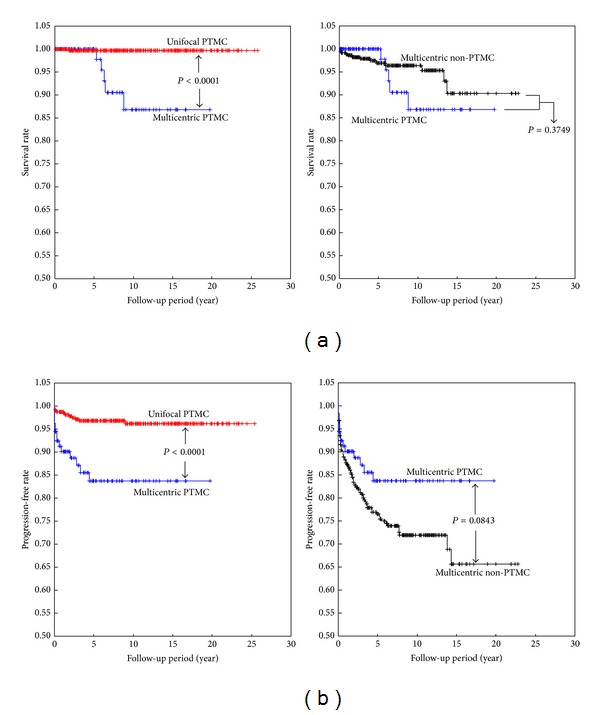
Cancer-specific survival curves (a) and progression-free rates (b) for multifocal papillary microcarcinoma and non-PTMC groups.

Cancer-specific survival and postoperative progression analysis were performed to compare multifocal PTMC with other types of PTC. The thyroid cancer-specific survival rates for unifocal PTMC and multifocal PTMC were 99.7% and 97.8% at 5 years; 99.7% and 86.8% at 10 years; and 99.7% and 86.8% at 20 years, respectively (Figure 2(a), left). Thyroid cancer-specific survival rates for multifocal PTMC and multifocal non-PTMC groups were 97.8% and 96.4% at 5 years; 86.8% and 95.3% at 10 years; and 86.8% and 90.3% at 20 years, respectively (Figure 2(a), right). Survival rate for multifocal PTMC was statistically lower than that for unifocal PTMC (*P* < 0.0001); in contrast, no difference was observed between multifocal PTMC and non-PTMC (*P* = 0.3749).

The PF rates for unifocal and multifocal PTMC were 96.8% and 83.7% at 5 years; 96.2 and 83.7% at 10 years; and 96.2 and 83.7% at 20 years, respectively (Figure 2(b), left). In addition, the PF rates for multifocal PTMC and multifocal non-PTMC tumors were 83.7% and 76.5% at 5 years; 83.7% and 71.9% at 10 years; and 83.7% and 65.6% at 20 years, respectively (Figure 2(b), right). The PF rate for multifocal PTMC was statistically lower than that for unifocal PTMC (*P* < 0.0001); in contrast, no significant difference in PF rate was observed between patients with multifocal PTMC and non-PTMC (*P* = 0.0843).

## 4. Discussion

Although the incidence rates of PTC and PTMC have increased in the last decade [2, 11, 12], the growing use of preoperative diagnostic modalities cannot explain this trend [11]. We have only recently gained sufficient information about the incidence of multifocal PTC in thyroid cancer patients to identify predictive factors. In this study, the incidence rates of both PTMC and multifocal PTC were found to have increased during the last 20 years. These increases highlight the need for further investigation of the clinical features, therapeutic outcomes, and long-term follow-up results of multifocal PTC in patients with microcarcinoma and larger tumors. More aggressive surgical procedures have been mentioned in recent papers [13, 14]. However, before reaching conclusions about alternative surgical treatments and postoperative ^131^I therapy, it is important to first obtain more information about the long-term follow-up results of multifocal PTC patients. Although our study data were collected from a single medical center, members of the thyroid cancer team, including those responsible for diagnoses, surgical approaches, and long-term followup, did not change during the last 20 years.

PTMCs that are incidentally diagnosed after surgery was performed to treat benign nodular lesions or Graves' disease usually have good prognosis, even in the absence of further postoperative treatment [15–18]. The Epidemiology and End Results Cancer Database identified 18,445 cases with PTMC during the period from 1988 to 2007 and concluded that the presence of 2 or more risk factors is strongly associated with cancer-related mortality [17]. However, multifocal lesions were not included in that analysis. In our study, patients with multifocal PTC had higher rates of lymph node metastases, soft tissue invasion, and distant metastases at the time of thyroidectomy. A more aggressive complete thyroidectomy with postoperative ^131^I ablation is indicated for such patients. Additionally, the effects of prophylactic central lymph node dissection for multifocal PTC must be investigated further [19]. To avoid transient or permanent hypoparathyroidism associated with total thyroidectomy, prophylactic central lymph node dissection ipsilateral to the tumor can be conducted. This approach may also improve the recurrence-free rate [20]. For cases with lymph node metastasis of bilateral multifocal PTC, concomitant contralateral paratracheal lymph node dissection may be indicated.

In our study, compared to unifocal PTC, multifocal PTC or PTMC was expected to correlate with higher recurrence rates or poorer prognosis. Among 483 multifocal PTC patients, there were 23 (4.8%) presented as distant metastases at the time of thyroidectomy. In addition, 23 of 98 (23.5%) postoperative progressive patients were presented with distant metastases. Additionally, total and disease-specific mortality rates were not increased in patients with multifocal PTC. More data and a longer follow-up period are needed to draw firm conclusions. Diverse mechanisms such as multiple independent tumors or intrathyroid spread originating from a single tumor mass were suggested for occurrences of multifocal PTC [5, 21]. In our study, the incidence of multifocal PTMC was not lower than that of multifocal non-PTMC. This finding indicates that the pattern of multifocal PTC manifests in early-stage thyroid cancer.

There is some controversy regarding the use of ^131^I ablation after total thyroidectomy to prevent recurrence of low- and intermediate-risk PTMC [22–24]. In our multivariate statistical analysis, we identified extrathyroid invasion, solid pattern, tumor multifocality, and absence of a tumor capsule as significant and independent risk factors for PTMC recurrence [25], although less information was available about postoperative ^131^I therapy for multifocal PTC. In our study, the ^131^I doses used to treat multifocal and unifocal PTC patients were not statistically different, and a higher rate of postoperative progression was noted in the multifocal group. Additional prospectively designed studies are required to determine the effect of higher ^131^I doses on preventing recurrence in patients with multifocal PTC.

Along with the study limitations described above, 17.3% of our patients did not undergo total thyroidectomy, and the thyroid remnant might have contained incidental microcarcinomas. Differences between PTMC and non-PTMC groups treated with total thyroidectomy may also represent a bias. Additionally, some of the patients did not receive ^131^I for remnant ablation.

In conclusion, multifocal PTMC occurred more frequently than non-PTMC. Additionally, postoperative disease progression and cancer mortality rates were higher in multifocal PTC than in unifocal PTC in both the PTMC and larger tumor groups. Furthermore, total thyroidectomy successfully reduced the postoperative disease progression rate in multifocal PTC patients with larger tumors.

## Figures and Tables

**Table 1:** Clinical features of multifocal papillary thyroid carcinoma (PTC) in microcarcinoma (PTMC) and non-PTMC groups.

	PTMC (*n* = 108)	Non-PTMC^#^ (*n* = 375)	Multifocal PTC (*n* = 483)	*P* value
Age (yr)	46.0 ± 11.9	45.5 ± 14.3	45.6 ± 13.8	0.7289
Female gender (%)	85 (78.7)	299 (79.7)	384 (79.5)	0.8153
Total or complete thyroidectomy (%)	91 (84.3)	363 (96.8)	454 (94.0%)	0.0001
Tumor size (cm)	0.7 ± 0.03	2.6 ± 0.1	2.2 ± 0.1	0.0001
TNM stage I (%)	92 (85.2)	213 (56.8)	305 (63.1)	0.0001
Postoperative Tg* (ng/dL)	161 ± 86	139 ± 32	143 ± 30.7	0.7663
^ 131^I dose accumulative dose (mCi)	150 ± 22.1	152 ± 11.2	151 ± 10.0	0.9583
Postoperative progression	14 (13.1)	84 (22.4)	98 (20.3)	0.0347
Follow-up period (yr)	5.3 ± 0.5	6.4 ± 0.3	6.1 ± 0.2	0.0503
Thyroid cancer mortality (%)	5 (4.6)	13 (3.5)	18 (3.7)	0.5679
Total mortality (%)	8 (7.4)	26 (6.9)	34 (7.0)	0.8652

Non-PTMC^#^: largest diameter of tumor over 1 cm; Tg*: serum thyroglobulin levels 4 to 6 weeks after thyroidectomy.

**Table 2:** Multifocal papillary thyroid carcinoma in postoperative progression and progression-free groups.

	Postoperative progression (*n* = 98)	Postoperative progression-free (*n* = 385)	*P* value
Age (yr)	47.1 ± 18.0	45.2 ± 12.4	0.2128
Female (%)	61 (62.2)	323 (83.9)	0.0001
Tumor size (cm)	2.8 ± 0.2	2.0 ± 0.1	0.0001
TNM stage I at diagnosis (%)	42 (42.9)	263 (68.3)	0.0001
Postoperative Tg* (ng/dL)	530 ± 132	38.8 ± 10.5	0.0001
Follow-up period (yr)	7.2 ± 0.5	5.9 ± 0.3	0.0277
Thyroid cancer mortality (%)	18 (18.4)	0	0.0001
Total mortality (%)	21 (21.4)	13 (3.4)	0.0001

Tg*: serum thyroglobulin levels 4 to 6 weeks after thyroidectomy.
